# Supplementary material for: Functional Impairment of Endothelial Colony Forming Cells (ECFC) in Patients with Severe Atherosclerotic Cardiovascular Disease (ASCVD)
Source: Int J Mol Sci. 2022 Aug 11;23(16):8969. doi: 10.3390/ijms23168969 (PMC9409296; doi:10.3390/ijms23168969)
Supplement: Supplementary file 1 [file ijms-23-08969-s001.zip › ijms-1842486-supplementary.pdf]

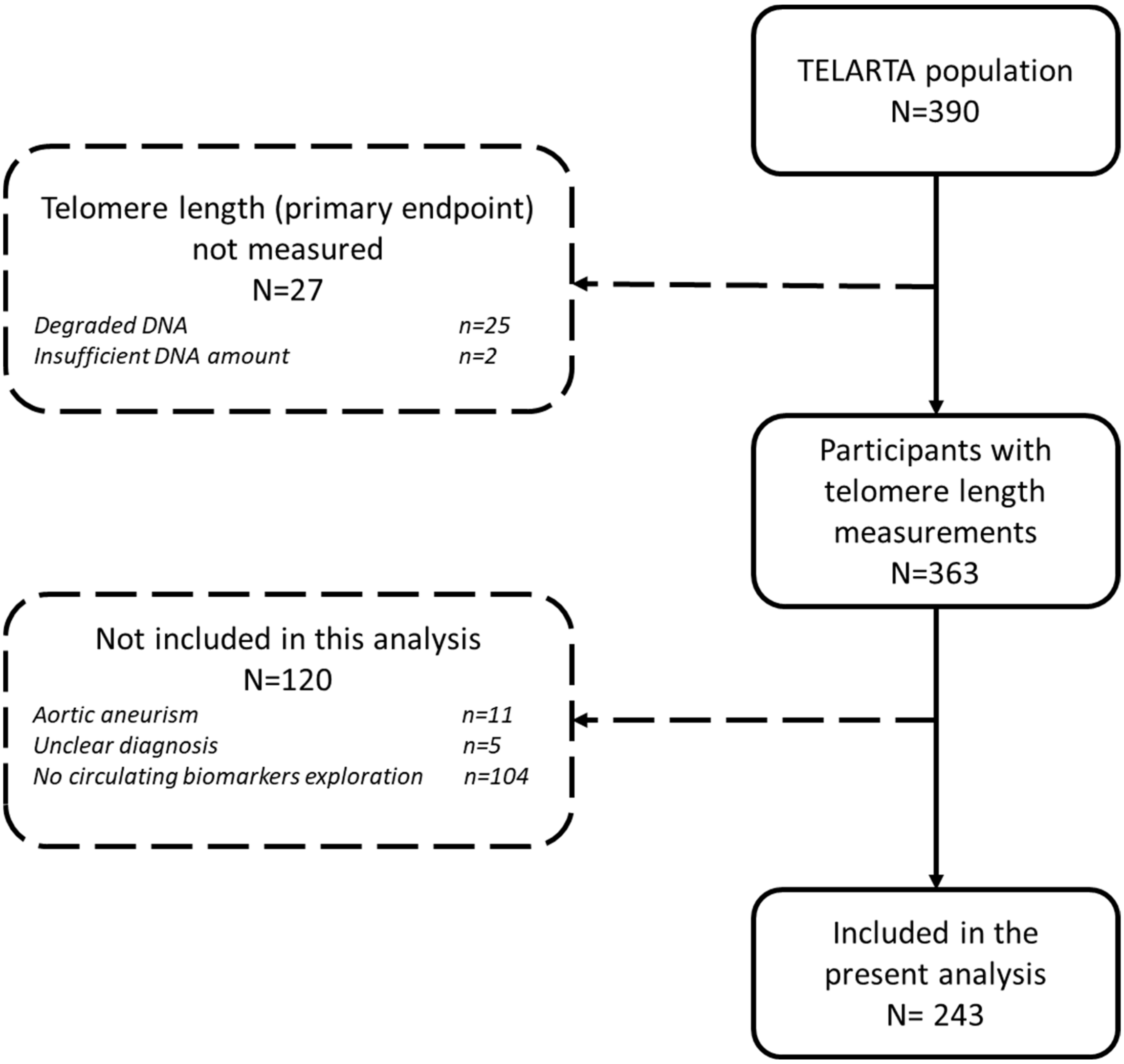

Figure S1: Flow chart of the participants included in the present analysis from the TELARTA project .

Participants in the dotted line boxes were excluded

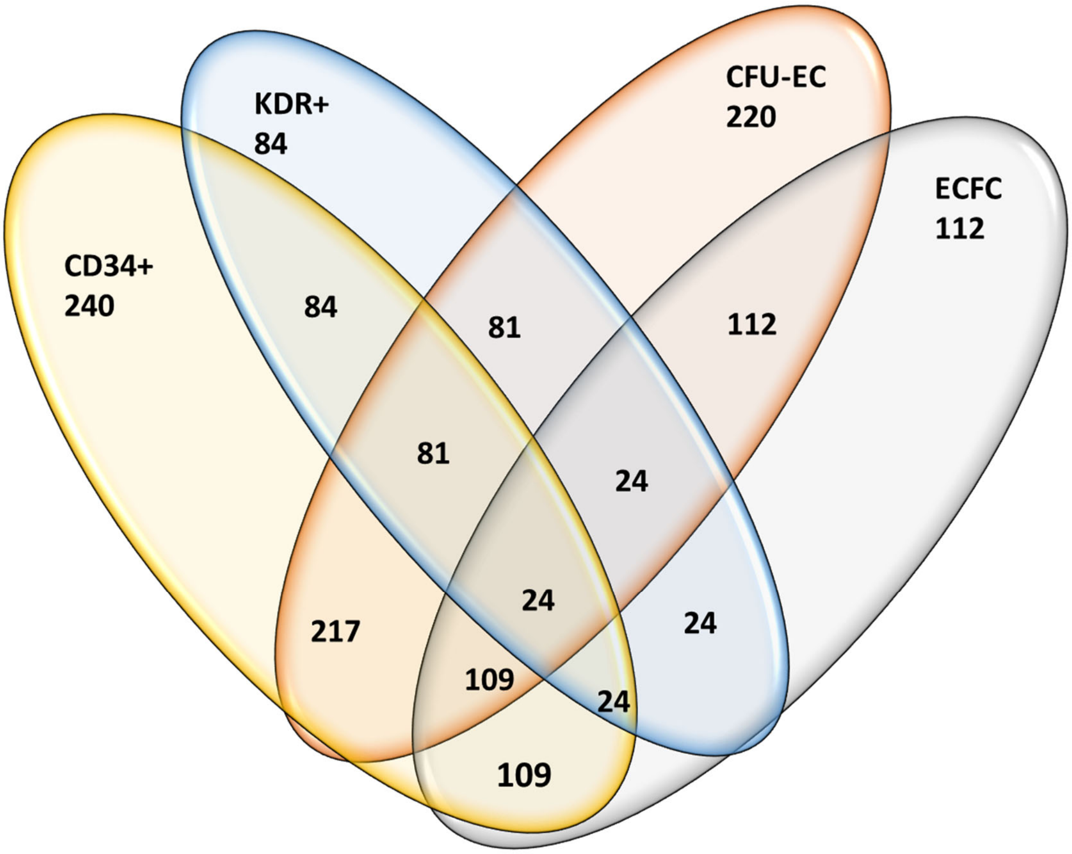

Figure S2 : Distribution of the participants according to the 4 different circulating biomarkers measured.

CD34+: circulating levels of CD34 expressing cells evaluated

KDR+: circulating levels of CD45, CD34 and KDR co-expressing cells evaluated

CFU-EC: Colony Forming Unit-endothelial cells (CFU-EC) capacity evaluated

ECFC: Endothelial Colony Forming Cells (ECFC) capacity evaluated
